# Risk Perception and Risk Communication for Training Women Apprentice Welders: A Challenge for Public Health Nursing

**DOI:** 10.1155/2013/386260

**Published:** 2013-10-30

**Authors:** Clarice Alves Bonow, Marta Regina Cezar-Vaz, Marlise Capa Verde de Almeida, Laurelize Pereira Rocha, Anelise Miritz Borges, Diéssica Roggia Piexak, Joana Cezar Vaz

**Affiliations:** ^1^Federal University of Pampa, 97501-570 Uruguaiana, RS, Brazil; ^2^School of Nursing, Federal University of Rio Grande, 96201-900 Rio Grande, RS, Brazil; ^3^School of Chemistry and Food, Federal University of Rio Grande, 96201-900 Rio Grande, RS, Brazil

## Abstract

This research has aimed to identify the perceptions of women apprentice welders about physical, chemical, biological, and physiological risk factors to which they are exposed and evaluate the identification of health disorders self-reported for women apprentice welders before and after implementation of a nursing socioenvironmental intervention. A quantitative study was performed with 27 women apprentice welders (first phase) and before and after an intervention with 18 women (second phase) in Southern Brazil in 2011. The data were analysed using SPSS 19.0. The participants identified the following risk types: physical (96.2%), chemical (96.2%), physiological (88.8%), and biological (62.9%). The results show a significant difference of the pre- and posttest averages for the musculoskeletal system and a posttest average increase for the integumentary, respiratory, and auditory system. A correlation of the women apprentices' ages and the identification of health disorders were made. It was understood that the perception of women apprentices regarding a particular set of occupational risks is essential for public health nursing to develop an effective risk communication as a positive tool for teaching and learning.

## 1. Introduction

This paper discusses the perceptions of women apprentices about the risks they are exposed to during welding activity. It also presents the development of a nursing socioenvironmental intervention as a tool for risk communication for health education of women apprentice welders. In different countries, for example, Nigeria [1], Sri Lanka [2], France [3], Denmark [4], Turkey [5], and Brazil [6], the issue involving the health and safety of welders is being discussed.

The motivation for the proposed research came from a literature review about the theoretical approach of risk perception [7–9]. On this occasion, the researchers observed the coherence and the need to analyse the issue of human risk in different environments, among these the apprenticeship environment, in relation to apprentices, which includes social, cultural, and political aspects in its production and reproduction [10–12]. Specifically, the interest in studying risk perception of apprentice welders is because the belief is held that the apprenticeship process represents a moment for health education, with the capacity of the dissemination of knowledge and the application of technology of public health nursing. In other words, during the apprenticeship, the apprentices should be encouraged to apply the knowledge learned about their health and on the future work environment. Besides this, it is believed that within this apprenticeship process, perceptions can be changed, from the comprehension of scientific knowledge to individual and collective behaviour, which can assist workers to produce healthy work environments. Primarily, the change or the creation of awareness about health, illness, and work can be enhanced in the apprenticeship process with the aim of directing the perception of what may or may not influence or even determine an injury, an illness, or better health conditions for workers and their work environment. Health education enables individuals to make informed decisions and adopt behaviour patterns that promote their health [13, 14]. 

The literature regarding apprentice welders shows concern about the achievement of improving welding techniques [15–17]. Specifically, in the area of health, the investigations include genetic disorders, respiratory problems, and exposure to metals. The first research concerned chromosomal aberrations in military apprentice welders in Aberdeen, MD, exposed to oxide ozone. Blood samples were collected from 273 apprentices for a period of 12 weeks. No statistically significant increases in chromosomal aberrations were found [18]. A cohort study aimed to determine the incidence of probable occupational asthma, bronchial obstruction, and hyperresponsiveness among 286 students entering an apprenticeship programme in the welding profession. The incidence of probable occupational asthma was 3% and of bronchial hyperresponsiveness 11.9%, defined as >3.2-fold decrease in the provocative concentration, causing a 20% fall in the forced expiratory volume in one second from baseline to the end of the study. These results show that exposure to gases and welding fumes is associated with changes in respiratory function [19]. However, a study sought to identify neuropsychological effects of low levels of exposure to manganese. Cognitive performance, motor control, and psychological tests were performed and assessed 46 apprentice welders at a local union welding school. Although the levels of manganese exposure were low, neuropsychological effects can become manifest, especially in relation to mood, attention, and fine motor control [20].

A search of the literature showed that there are texts that present apprentice welders as subjects, covering the welding technique and health of the subjects. The improvement of welding techniques contributes to the reduction of accidents during this activity, as it also does regarding possible injuries and accidents as a result of welding activity. However, there were no texts that show risk perception related to the activity of apprentice welders and the concern with the apprenticeship process about health and safety at work, from theories of risk perception and risk communication because of the approaches necessary for system management of risk to human health at work. There were also no texts observed which identify women as subjects of this occupational activity. 

According to the theoretical orientation, this research assumed that the notion of risk perception involves two factors: the magnitude of potential loss and the probability of its occurrence [21]. In other words, the existence or not of different risk factors and occupational accidents might explain why people perceive the same risk in very different situations or why the same individual might perceive risk differently, depending on when he or she is asked about it [22]. 

Risk perception encompasses both personal and work-environment-related ideas and constructions because, to perceive it, you have to believe it [21]. Therefore, the study of apprentice welders' risk perception is important, as individuals are responsible for the risks perceived in the work environment and that individual might have caused the risk which an individual perceives. This creates the possibility of changes to minimize or even eliminate risk factors related to individual behaviour or even to their own working conditions. One of the processes of interaction to promote the various changes may be the tool of risk communication.

Risk communication is here understood as an interactive process of exchange of information and opinion among individuals, groups, and institutions [23]. Risk communication can also help promote changes in individual and collective behaviour. Risk communication theory and practice may include public participation and conflict resolution. Risk communication, as aforesaid, was used as a tool for the development of a nursing socioenvironmental intervention with apprentice welders. For this study, the results of an intervention with women apprentice welders are described.

Another theoretical orientation is a classification of different risk factors that the apprentice welders are exposed to. Therefore, the Act of 16 June 1999 [24] was used, which provides occupational hygiene and safety standards and the obligations of employers and employees to create a safe work environment, organization of hygiene and safety at the level of the enterprise, institution, and state, procedures for settlement of disputes in this matter, and responsibility for breaches of established standards. In the specific case of apprentice welders, during welding activities they are exposed to various occupational risks generated by chemical, physical, biological, and physiological risk factors. 

These factors can create or worsen occupational health disorders. Among some of the health disorders that may be triggered due to welding activity are cited burns on the skin, which can cause skin cancer [25–27], lung cancer [28, 29], stomach cancer [30], coronary heart disease [31], noise-induced hearing loss [32, 33], and cumulative trauma disorders [34].

The physical risk factors that welders are exposed to include noise from welding machines and nonionizing radiation from open welding arcs. Such factors may trigger disturbances related to the auditory system [32, 33] and the integumentary system, such as skin cancer [25–27]. Skin cancer can be related to frequent skin burns suffered by welders [35]. Burns originate from hot metal contact, which become chemical burns. Thus, chemical burns are related to physical risk factors (heat) and chemical risk factors (different chemical compounds present in the metals that come in contact with the skin). 

Chemical risk factors still include contact with different metals in a gaseous state. Risk factors from exposure to welding fumes include chemical contact with different metals. Exposure to welding fumes from the burning of these metals can cause respiratory disorders. An example of a harmful compound is stainless steel, the smoke of which can cause acute lung injury and the size of the inhaled particles and exposure time are significant factors in welding, which must be considered in the development of protective strategies [28]. Another example is exposure to chromium. A cohort study performed with male welders in the period from 1964 to 1984 identified a higher incidence of lung cancer [29]. 

Besides the respiratory system, exposure to chemicals also exposes welding workers to disturbances in their cardiovascular systems. Research conducted with construction workers, and this included welders, indicated heart rate variability during occupational exposure and also at night, showing that inhaled metal particles during work have an organic influence, specifically causing arrhythmias [31]. Another system that can be damaged due to chemical risk factors is the gastric system. The profession welders are at risk of stomach cancer, due to working in dusty environments [30]. 

Physiological risk factors include poor posture during welding because workers perform the activity with a flat piece of metal and they must move around the piece to make the weld. This feature demands that the employees remain in an ergonomically incorrect posture in order to obtain a better result of the weld. Furthermore, excessive vibration from the welding machine is associated with back pain [34]. 

For these reasons, the present study has aimed to identify the perceptions of women apprentice welders about physical, chemical, biological, and physiological risk factors to which they are exposed and evaluate the identification of health disorders self-reported for women apprentice welders before and after implementation of a nursing socioenvironmental intervention. 

## 2. Materials and Methods

### 2.1. Design

This study consists of two phases. The first phase is a quantitative, exploratory, and descriptive study involving women apprentice welders, conducted in 2011 in Rio Grande (Rio Grande do Sul, Brazil). The second phase consists of a quasi-experimental, nonrandomized study, which was made before and after nursing socioenvironmental intervention as a tool for risk communication for women apprentice welders enrolled in this study, using the results obtained in the exploratory study (first phase), conducted in 2011 in the same region.

This study is part of a larger research project entitled “*Health, Risks and Occupational Diseases: An Integrated Study in Different Work Environments*” [36]. It was approved by the Research Ethics Committee of the Federal University of Rio Grande (Universidade Federal do Rio Grande—FURG). Women apprentice welders were included in the study after signing an informed consent agreement. The study was conducted using public funds (National Counsel of Technological and Scientific Development—CNPq) and linked to the Laboratory of Socio-environmental Process Studies and Collective Production of Health (LAMSA) research group of the Nursing School of the Federal University of Rio Grande.

### 2.2. Sample

 The sample of subjects, intentional nonprobabilistic, was composed of 27 women apprentice welders (first phase) enrolled in the technical programme for training as welders in Rio Grande, Rio Grande do Sul, Brazil. Women apprentice welders were divided into eleven classes. The total number of apprentice welders was 162. Women apprentices represented 16.6% of the total number of apprentice welders. 

For the second phase, consisting of a nursing socioenvironmental intervention as a tool for risk communication, six classes (86 apprentice welders; 18 women apprentices) were invited, all of which participated in the first phase. In addition to the apprentice welders, six members of the research group LAMSA also participated, as mediators of the nursing socioenvironmental intervention. The welding course which the apprentices were doing includes theoretical and practical lessons. The classes in which nursing socioenvironmental intervention activities were applied had already started practical lessons. 

### 2.3. Measures

The first phase of the study was conducted, based on the following question: how do women apprentice welders perceive the risks to which they are exposed? From the theoretical basis assumed in the study, the existence of a relationship between risk perception and identification of health disorders self-reported by women apprentice welders was identified. Data collection was performed in 2011, through a structured interview from a questionnaire, composed of mixed questions—multiple-choice and single-choice. 

The structured questionnaire had multiple-choice and single-choice questions corresponding to the following variables: participant characteristics (age, skin colour/ethnic origin, level of schooling, and marital status); time of experience in welding; risk perception among apprentice welders (the identification of chemical, physical, biological, and physiological risk factors).

Upon the completion of the first phase of the research, the authors organized a nursing socioenvironmental intervention in the study group (second phase). The results of the first phase were used to develop risk communication concerning the risk factors of the work environment as an apprenticeship tool to help apprentice welders for the promotion of individual and collective health in the workplace. Only data from nursing socioenvironmental intervention with women apprentice welders will be presented in this study. 

After analysing data from the first phase, the issues to be developed during the nursing socioenvironmental intervention with apprentice welders were organized. Six apprenticeship workshops were conducted, each with a group of apprentice welders. The time used for planning was 40 hours. Four hours were allocated for holding each apprenticeship workshop, making a total of 24 hours. The apprenticeship workshops took place in the theoretical room of the institution. 

This practice also included the Health Promotion in Different Work Environments Programme (HPDWEP) [37, 38] of LAMSA, the School of Nursing, the Federal University of Rio Grande, RS, Brazil. The HPDWEP consists of a set of coordinated actions and continuous shaft in promoting social and environmental health in different work environments, the environments of which are included in the study group's academic LAMSA.

The nursing socioenvironmental intervention was developed, based on the risk communication concept [7–9, 23, 39]. The content (message) about the nature of risk was developed through the classification of risk factors (physical, chemical, biological, and physiological) and health disorders, due to exposure to these risk factors, based on the Occupational Safety and Health Act of 16 June 1999 [24] of the International Labour Organization (ILO).

Physiological systems were approached in the nursing socioenvironmental intervention in the following order: integumentary, respiratory, cardiovascular, auditory, musculoskeletal, and gastric. Anatomic-physiological systems, risk factors present in welding activity, and health recommendations for apprentice welders were presented to the apprentice welders. 

**Figure 1:**
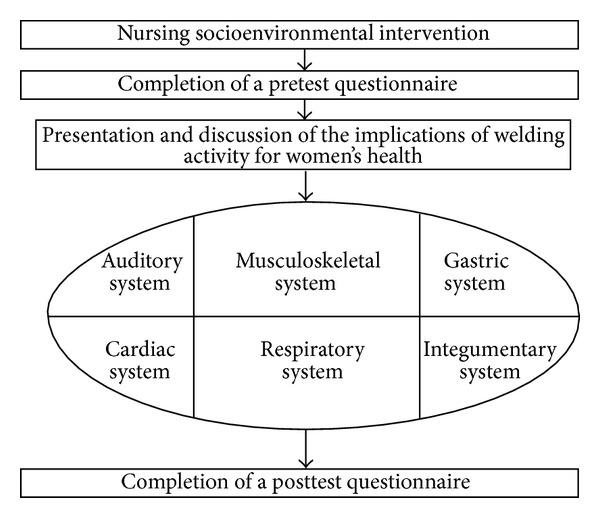
Steps of nursing socioenvironmental intervention.

The nursing socioenvironmental intervention used the following steps: (1) presentation of the study and research group and the signing of an informed consent agreement; (2) completion of a pretest questionnaire; (3) implementation of the nursing socioenvironmental intervention; (4) completion of a posttest questionnaire (Figure 1). The last step always occurred on the last day of the welding course. During the nursing socioenvironmental intervention it was possible to relate risk factors that the apprentice welders are exposed to and physiological systems which are affected by these. 

To trigger the development of communication (first step) with the apprentices who were participating in the intervention, the following question was used: what personal protective device is used during welding activity? The responses were expressed on a whiteboard for all the apprentices to see. The answers were welding cap, welding apron, welding coat, welding boots, earplugs, welding trousers, welding goggles, welding mask, breathing mask with filter, and welding gloves. This promoted the apprentices to make comparisons, considerations, and suggestions on the subject. There were comparisons about the personal protective device used by the apprentices because some only use the welding coat and trousers (provided by the technical programme for training) and others use items not included in the personal protective device supplied by the technical programme for training, for example, the welding apron (individual purchase), in order to increase protection. Moreover, some apprentices do not use the breathing mask with filter because it is uncomfortable, which generated discussion among the participants of the apprenticeship workshops. 

To continue the process of risk communication, visualization of personal protective devices used to perform welding activity made it possible to show the different body systems (integumentary, respiratory, and auditory) which are protected by personal protective devices. Besides these, the gastric, cardiovascular, and musculoskeletal systems were included, which, despite not being protected by personal protective devices, require attention during welding activity. 

During the presentation of the integumentary system, concerns about the physical risk factor, nonionizing radiation, and chemical risk factors, due to frequent skin contact with metals, were focused upon. Apprentices were asked about the composition of the wire used to perform the welding. They use a wire called E71T-1, which is composed of carbon, manganese, silicon, phosphorus, and sulphur. It was emphasized that every time the apprentices have skin contact, either by touching the metal or through weld splash, they are in contact with heavy metals and minerals present in the wire, especially when the skin is hit by a weld splash because, due to its elevated temperature, the splash causes chemical burns. It was recommended to use sunscreen, especially during welding activity and when exposed to solar radiation, and also to use welding gloves during activity followed by proper hand washing in order to minimize contact with metals. 

To explain the proper hand washing method, a poster was devised by LAMSA. During the explanation the gastric system was discussed because the apprentice welders can ingest metals when they eat if proper sanitation of their hands is not performed after working with the solder.

Concerning the respiratory system, chemical risk factors were dealt with which apprentices are exposed to because they breathe the fumes resulting from the burning of metals during welding activity. The composition of the wire E71T-1 was again referred to explore the importance of a breathing mask with filter, a respiratory mask with filter being provided by the technical programme for training, which protects against dust and fumes from welding. It is important to use a breathing mask with filter under the welding mask because without it the welders will be inhaling dust and fumes from the welding process. Besides the chemical compounds present in fume welding, apprentices are also in contact with gases (acetylene and carbon dioxide) that are released during the opening of the flame. Unfortunately, the mask provided does not protect against inhalation of gases. For these reasons, it was recommended that apprentices do not remain in the environment of the welding practice rooms unnecessarily and/or without the protection of the respiratory mask with filter. Physical activities were recommended that promote breathing, such as running races, in order to encourage gas exchange, which also promotes the health of the cardiovascular system. 

For the auditory system approach the physical risk factor was noise. The apprentices were informed about exposure to 89-90 dB from the welding machine during practical activities. During the practical classes of each class about 14 welding machines are used. The noise is caused by exhaust fans, which exceed the limit of 115 dB, which is the imposed limit for occupational exposure without proper protection, according to Regulatory Standard 15 from Brazil [40], which provides tolerance limits for continuous and intermittent noise. In addition, most apprentices use earplugs, such as headphones, which, unlike earmuffs, offer less protection than earplugs. Apprentices were questioned on how they perform ear cleaning during the activities and practices of welding and on shared earplugs among apprentices. Some apprentices reported not performing ear cleaning and that they never lent earplugs. Daily cleaning with soap and water for earplugs was recommended and advice was reinforced about not lending earplugs because of the ease of transmission of bacteria by this route. 

For the musculoskeletal system, the following physiological risk factors were approached: performing repetitive movements, staying in the same posture for long periods and sometimes incorrect posture, and risk factors which apprentice welders are exposed to. To minimize exposure to these risk factors, the apprentices were asked to perform stretching exercises. During the exercises, apprentices were instructed to carry out the activity of stretching before and after welding practice and at intervals of 10 minutes after 50 minutes of welding activity. Physiological systems, risk factors, and health effects during welding activity are presented in Table 1. 

In addition to these recommendations, after exposure of the systems, the following general recommendations were made: prioritize foods rich in iron and calcium to promote the excretion of manganese, prioritize foods rich in vitamin C to facilitate iron absorption, and prioritize food rich in fiber to facilitate removal of manganese and other metals by feces, since only some of the manganese is eliminated in the urine. 

To continue risk communication, the results of this research were presented. This approach focused on reestablishing the perception of risk factors (physical, chemical, biological and physiological) to which apprentices are exposed and health disorders related to welding activity. The presentation was concluded with the delivery of explanatory posters, which were placed in the welding practice rooms, so that by looking at the poster the implementation of protective measures during welding activity and the minimization of exposure to risk factors would be stimulated. 

The pre- and posttest questionnaire consisted of 41 variables related to identification of health disorders: musculoskeletal (15 items), integumentary (12 items), auditory (4 items), gastric (4 items), respiratory (3 items), and cardiovascular (3 items). The answers were given on a Likert Scale of five points, with the lowest being 0 (never feel/felt it) and the highest 4 (always feel/felt it). Thus, the maximum average of each block of questions was four. 

### 2.4. Data Analysis

 The Statistical Package for Social Sciences (SPSS) software Version 19.0 was used to organize and describe analysis of the data (first phase). Data from nursing socioenvironmental intervention (second phase) was presented using percentage, mean, and standard deviation (±SD). For paired samples analysis, were used student *t* test (*P* < 0.05). The Spearman correlation was used to analyse the intensity of the relation between the variable age, time of experience in welding, and self-reported health disorders by women apprentice welders before and after nursing socioenvironmental intervention.

## 3. Results

The sample included 27 women apprentices enrolled on the technical programme for training as welders in Rio Grande, Rio Grande do Sul, Brazil. Their ages ranged from 18 to 56 years, with an average of 30.26 years (±8.39); 11 (40.7%) were ethnically white and 11 (40.7%) ethnically black; 19 (70.4%) were single; 14 (51.9%) had finished secondary school and 12 (44.4%) had no children (Table 2). Regarding time of experience in welding, 20 (74.1%) had none, 6 (22.2%) had experience, and one (3.7%) did not answer the question on experience. The average of time of experience ranged from 4 to 24 months, with a mean of 10.67 months (±7.52).

The results of the questionnaire on risk perception in the welding apprenticeship environment showed that 26 (96.2%) women apprentice welders identified physical risks, 26 (92.2%) identified chemical risks, 24 (88.8%) physiological risks and 17 (62.9%) biological risks. Among the risk factors identified, the most frequent was the heat during welding activity and the presence of gases, cited by 21 (77.8%) women apprentice welders (Table 3). 

Table 4 presents mean (±SD) identifying organic disorders in different times (before, after, and in relation to between before and after nursing socioenvironmental intervention). The musculoskeletal and integumentary systems had the highest averages, demonstrating greater identification of women apprentice welders about health disorders in these systems. 

Comparing the means before and after nursing socioenvironmental intervention, it can be seen that there was an increase of the auditory, musculoskeletal, respiratory, and integumentary means. This increase of averages indicates that there was identification of disorders in these systems after nursing socioenvironmental intervention. The evaluation after the nursing socioenvironmental intervention shows a decrease in the mean cardiovascular and gastric systems, which points to a lower reference of women apprentice welders to disorders related to these systems. 

The *t-*test detected a higher difference between means before and after nursing socioenvironmental intervention in the musculoskeletal system (*P* < 0.05). For the other systems there were no significant differences. 

Spearman correlation between age, time of experience, and self-reported health disorders by women apprentice welders revealed a negative correlation between age of women apprentices welders and the average after intervention for the gastric system (*P* < 0.01); that is, the lower the age, the higher the average during the valuations of such systems. The pattern changes when the correlation between organ systems is analysed, indicating among most of them positive and significant correlation. Time of experience was not correlated with any variable. 

## 4. Discussion

This study contributes to an understanding of the perception of risk factors and identification of health disorders self-reported by women apprentice welders. The identification of the risk factors perception contributes to health education for risk communication, as in the case of nursing socioenvironmental intervention. Health education is an important strategy to prevent diseases [14]. 

As regards the perception of risk factors that were identified, risks were reported in decreasing order: physical, chemical, physiological, and biological. Regarding health disorders self-reported by women apprentice welders, the average for before and after nursing socioenvironmental intervention was higher for health disorders related to the musculoskeletal and integumentary systems, indicating greater identification of women apprentice welders in relation to welding work and health disorders. Furthermore, the greater identification of musculoskeletal and integumentary disorders is associated with more perceived risk factors (physical, chemical, and physiological).

It should be emphasized that with the *t*-test the musculoskeletal system showed significant difference. These findings are similar to those found in the literature on welding-work-related disorders, which present the welders as a group at risk for musculoskeletal and integumentary disorders [25–27, 34].

The physiological risk was reported by 88.8% of women apprentice welders, showing mainly poor posture and repetitive stress. The postures, repetitive movements, and constant vibration of the welding machines are examples of wear suffered by the musculoskeletal system. Most activities in welding require a variety of movements, such as bending, stretching, and long periods of standing, and, to perform these activities, specific muscle groups are used, such as the lumbar and scapular muscles, resulting in overload and increase of the risk of disorders [41]. In addition, physiological risk factors are present in other work environments, such as temporary dock work, which can trigger work-related musculoskeletal disorders [42].

Studies [43, 44] were performed because of concern about manual labour in relation to the constant vibration of the tools of the welders during the welding process. The investigation found that the tools exceed the exposure limits when operated for more than 8 hours. Research [34] performed with different workers showed that, specifically for welders, vibration may be associated with back pain.

It is important to highlight that musculoskeletal disorders associated with welding activity may occur due to the need for constant physical effort of women apprentices and future workers. To be specific, a significant difference to the musculoskeletal system can be explained by the fact that the pains arising from disturbances in this system presented multifactorial origins, related to work, the individual worker characteristics, personality traits, and life history [45]. Thus, the results of the pretest identified the association; however, after the nursing socioenvironmental intervention, the association was greater for identifying how the activity is related to welding disorders in the musculoskeletal system.

**Figure 2:**
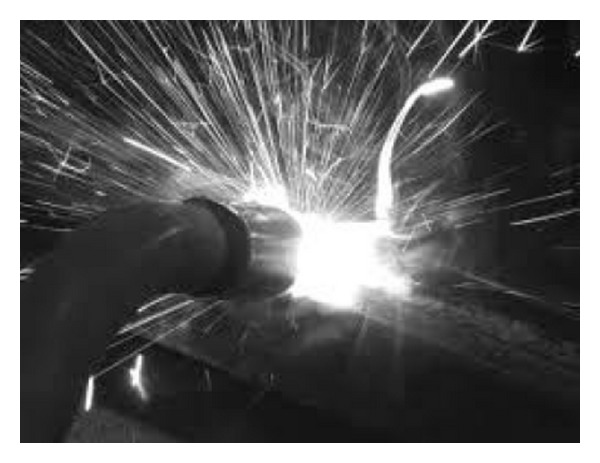
Opening for electric arc welding activity.

Concerning physical risks, they were identified by 96.2% of the study participants. This is due to the constant exposure of women apprentice welders to weld spatter and hot metal objects, according to the activity they perform. This frequent exposure can cause a greater number of disorders related to the integumentary system. The main risk identified was physical heat for 77.8% of the participants. The heat self-reported by women welding apprentices arises from the nonionizing radiation produced by welding activity. More specifically, heat is produced during the opening of the electric arc (Figure 2), which consists of an electric discharge.

Study findings show that the intensity and wavelength of nonionizing radiation produced would depend on many factors, such as the type of welding process, welding parameters, and the composition of metals, fluxes, and any coatings that may be on the base material. Moreover, the radiation exposure time was considered compatible with each 8 hour exposure within a 24-hour period. Therefore, two exposures of 5 minutes during a workday can be considered as a single 10-minute exposure. The research results show that the minimum safe distance for 1 minute is 32 cm [35].

Another study [46] conducted to quantify the risk of arc eye during welding activity showed that the maximum exposure without protection is around 0.47 to 4.36 seconds. For this reason, it is important that welders avoid direct exposure to light to initiate the welding arc. Moreover, they must use personal protective devices appropriate for the eyes and for the type of weld. 

The integumentary system is mainly exposed to ultraviolet (UV) radiation coming from the open arc welding activity. Occupational exposure to UV increases the risk of skin cancer [24–26]. An example is presented in a case study [27] situation in which sequential bilateral ocular melanoma is reported in an electric arc welder with 15 years of work. The authors associate the presented patient's predisposition with cancer as being due to his occupational activity. A case-control study [47] conducted with people diagnosed with ocular melanoma showed no increased risk of this cancer in the groups exposed to UV radiation at work, as in the example of welders. 

Examples of integumentary disorders that may be caused by physical and chemical risks present in welder's activity are occupational burns. Occupational burns are divided into three categories. Thermal burns include events that result from high levels of heat caused by explosions, flame, radiant heat, and direct contact with hot surfaces. Electric injuries result from electrical explosions, flashes, or direct contact with an electrical current. Chemical burns result from the reaction of biological tissue with chemical materials [48]. 

Specifically with apprentice welders, burns that can occur include thermal burns and chemical burns. There is a study which describes the occurrence of work-related injuries from thermal, electrical, and chemical burns among electric utility workers, among these the welders. Welders (not a common occupation in the electric utility workforce) had the highest age-adjusted injury rates for all burn-related injuries (61.57 per 10,000 employee-years) and for thermal/heat burns (40.87 per 10,000 employee-years) [49]. It is understood that in the case of welding activity, a thermal burn may constitute a chemical burn, as contact with the chemical compounds present in the metal that cause thermal burns can causes a chemical burn. 

Another important issue is related to exposure to radiation from welding machines. The effects of exposure to such radiation were tested on male and female welders who are parents and who are exposed to magnetic fields. The investigation was performed in order to detect an increased risk of cancer in the children of these workers. The association between these factors has not been proven [50]. Likewise, an experimental study [51] conducted with rats during the gestation period involving exposure to radiation showed that although there was no teratogenicity there were problems of poor bone formation and low birth weight.

The findings also suggest that the perception of chemical risk identified by 96.2% of the participants and the identification of health disorders involving this risk, for example, respiratory and integumentary disorders, increased after nursing socioenvironmental intervention. This risk perception, related to the chemical risk and identification of health disorders related to the chemical risk, is related to the raw material that the apprentices handle during welding activity, for example, the hot metal [52]. The metals which apprentice welders are in contact with include aluminum [53], stainless steel [29], cadmium [54], chromium [55], lead [56], copper [57], manganese [57], molybdenum [57], and nickel [56]. These chemicals may generate hazardous fumes during welding activity. According to the International Labour Organization [52], these metals are related to risk factors and the occurrence of health disorders, when the welders are hit by weld splash or hot metal particles or inhale metal fumes (respiratory health disorders).

Among the chemical risks, 77.8% of women apprentice welders recognized the gases with which they deal during welding activity as risk factors, 59.3% identified the dust present in the apprenticeship environment, and 29.6% the fumes from welding. Research indicates that the welding fumes from the chemical compound, stainless steel, can cause acute lung injury and the size of the inhaled particles and exposure time are significant factors in the welding, which must be considered in the development of protective strategies [28]. Lung function and respiratory symptoms in welders were therefore investigated in a case-control study [58], noting significantly higher prevalence of respiratory symptoms (dyspnea and secretion) in welders. The study suggests that the welders are at risk of developing respiratory symptoms and decreased lung function, although the concentrations of metal fumes were lower than the recommended limit by the American Conference of Industrial Hygienists (ACGIH).

Another important pathology in welders is lung cancer. A cohort [29] conducted with male welders, from 1964 to 1984 showed that the incidence rate of lung cancer was higher. An important chemical compound, carcinogen, found in welding activity, is chromium. Studies suggest that chronic occupational exposure during welding activity can raise levels of damage to genetic material and inhibit the repair of the same [55, 59]. 

A longitudinal study of apprentice welders showed a significant association between welding-related metal fume and respiratory symptom fever as well as a decrease in lung function values after 15 months in welding school [19].

Analysing the average of the remaining systems obtained in the pre- and posttest, it was noted that there was an increase in the average posttest for the auditory and respiratory systems and a decrease in the average for the cardiovascular and gastric systems. It can therefore be considered that the provision of a nursing socioenvironmental intervention for women apprentice assists in the dissemination of knowledge to identify health disorders related to welding work. 

By identifying these results, the conclusion will be the idea that risk communication strategies, as is the case with nursing socioenvironmental intervention, enable different groups to identify sources of information and knowledge, which are specific instruments in the role of health protectors of apprentices and future workers, as well as their colleagues. It should also be noted that by relating characteristics of the work and the conditions of exposure to different risks related to possible disorder, the apprentice can become proactive in the protection of her health.

A study with different groups of community residents in the United States but with similar exposure problems examined the presence of manganese in the air and identified the community perception of the local air quality and the effects of manganese exposure on health. Through this identification, the authors used the risk communication strategy for teaching/learning self-care for this group. The results of risk communication showed a strong link with the academic community responsible for this area. However, such a result was possible due to the type of risk communication established, which visualized the needs and experiences of the community, integrating this knowledge with research protocols [60]. Similar studies with female welding apprentices were not found.

The correlation between age and the results of the posttest for the gastric system indicates that the youngest women apprentice welders identified more disorders related to the systems than apprentices who were older. It is understood that the opportunity to relate the health disorders with welding activity during the nursing socioenvironmental intervention provided by nurses was seized by the younger welders. This result was also evidenced in research that sought to explore the impact of an online learning theoretical course of welding and electrics, where younger learners had higher levels of compliance [61]. The condition of apprehending knowledge of younger people may be a factor that contributes to this correlation. Older apprentices have formed concepts about certain subjects, which can trigger greater difficulty for apprehending different knowledge from that which has already been acquired.

## 5. Conclusions

In conclusion, women apprentice welders realize that they are exposed to risk factors, especially chemical and physical risk factors related to the fact that their workplace is particularly dangerous. Results showed that the nursing socioenvironmental intervention provided information about health disorders related to welding activity for the auditory, cardiac, gastric, musculoskeletal, respiratory, and integumentary systems. Such information enabled the women apprentice welders to evaluate information and through this self-report health disorders. Analysis of the mean pre- and posttest of the musculoskeletal system allowed for observation of the influence of nursing socioenvironmental intervention on the apprehension of women apprentices of health disorders knowledge related to welding activity. 

In order to minimize musculoskeletal health disorders related to welding activity, the recommendation is made that women apprentices should perform stretching activities before and after work and must perform ten-minute breaks every 50 minutes of work. Besides musculoskeletal disorders, other disorders, for example, integumentary disorders, such as skin cancer, can be prevented through simple measures, as through the use of sunscreen during welding activity; respiratory, gastric, and cardiac disorders, such as lung and stomach cancer and cardiac arrhythmias, can be prevented by the use of a respirator, doing physical activities in order to facilitate gas exchange, and washing hands after working with weld to prevent ingestion of metals; and auditory disorders, such as noise-induced hearing loss, can be avoided through the use of hearing protection during the whole period of working with weld.

It is believed that risk communication, through a process of health education, can modify individual behaviour because it is a process in which apprentices perceive and multiply knowledge in their work/apprenticeship environment and thus interfere with collective work conditions. It is understood that the perception of women apprentices regarding a particular set of occupational risks is essential for public health nursing to develop an effective risk communication as a positive tool for health education.

## Figures and Tables

**Table 1:** Physiological systems, risk factors, and health effects during welding activity.

Physiological systems	Risk factor	Health effects
Integumentary	Physical and chemical	Chemical burns Skin cancer
Gastric	Chemical	Stomach cancer
Respiratory	Chemical	Lung cancer Pneumonia Occupational asthma
Auditory	Physical	Noise-induced hearing loss
Musculoskeletal	Physiological	Work-related musculoskeletal disorders
Cardiac	Chemical	Cardiac dysrhythmia

**Table 2:** Demographic characteristics of study subjects (*n* = 27).

Variables	Categories	*n*	Percent (%)
Marital status	Single	19	70.4
	Married	5	18.5
	Separated	2	7.4
	Widowed	1	3.7

Skin colour/ethnic origin	White	11	40.7
	Black	11	40.7
	Brown	5	18.5

Schooling	Elementary school, incomplete	1	3.7
	Elementary school	3	11.1
	Secondary school, incomplete	5	18.5
	Secondary school	14	51.9
	Higher education, incomplete	2	7.4
	Higher education	1	3.7
	Postgraduate education, incomplete	1	3.7

Number of children	None	12	44.4
	One	5	18.5
	Two	3	11.1
	Three	4	14.8
	Four	2	7.4
	More than four	1	3.7

**Table 3:** Perception of women apprentice welders about physical, chemical, biological, and physiological risk factors (*n* = 27).

Risk factors	*n*	Percent (%)
Physical		
Heat	21	77.8
Noise	19	70.4
Ionizing radiation	13	48.1
Nonionizing radiation	4	14.8
Abnormal pressures	4	14.8
Moisture	4	14.8
Vibrations	3	11.1
Cold	2	7.4
Chemical		
Gases	21	77.8
Dust	16	59.3
Chemical products	15	55.6
Fumes	8	29.6
Vapours	6	22.2
Mist	2	7.4
Fog	1	3.7
Biological		
Bacteria	8	29.6
Fungi	8	29.6
Bacilli	4	14.8
Virus	2	7.4
Parasites	2	7.4
Protozoa	2	7.4
Physiological		
Poor posture	17	63.0
Repetitive strain	12	44.4
Inadequate ventilation	10	37.0
Use of inappropriate equipment	9	33.3
Rhythm of overwork	7	25.9
Machines and/or inadequate equipment	5	18.5
Requirement productivity	3	11.1
Inadequate lighting	2	7.4

**Table 4:** Percentage change in average of identification of health disorders self-reported for women apprentice welders before and after nursing socioenvironmental intervention (*n* = 18).

Health disorders	Evaluation					*P *
	Before		After		Before/after	
	*n*	Mean (±SD)	*n*	Mean (±SD)	mean (±SD)	
Auditory system	18	0.30 (0.46)	16	0.32 (0.40)	−0.03 (0.32)	0.708
Cardiovascular system	18	0.29 (0.58)	16	0.22 (0.37)	−0.02 (0.14)	0.580
Gastric system	18	0.33 (0.68)	16	0.28 (0.39)	−0.04 (0.40)	0.646
Musculoskeletal system	18	0.64 (0.57)	16	1.03 (0.52)	−0.43 (0.58)	0.010
Respiratory system	18	0.27 (0.44)	16	0.49 (0.61)	−0.24 (0.50)	0.068
Integumentary system	18	0.69 (0.46)	16	0.78 (0.48)	−0.08 (0.45)	0.491
